# Neonatal malaria management: a global survey of current practice

**DOI:** 10.7189/jogh.16.04367

**Published:** 2026-11-06

**Authors:** Ayten Sultanli, Jonathan Remppis, Yabo Josiane Honkpehedji, Carolin Reif, Meral Esen, Ayola Akim Adegnika, Sabine Bélard

**Affiliations:** 1 Institute of Tropical Medicine, University of Tübingen, Tübingen, Germany; 2 German Centre for Infection Research (DZIF), Partner Site Tübingen, Germany; 3 Institute for Clinical Epidemiology and Applied Biometry, Faculty of Medicine, Eberhard Karls University Tübingen, Tübingen, Germany; 4 General Paediatrics, Paediatric Neurology and Developmental Medicine, University Children's Hospital Tübingen, Tübingen, Germany; 5 Centre de Recherches Médicales de Lambaréné (CERMEL), Lambaréné, Gabon; 6 Leiden University Centre for Infectious Diseases (LUCID), Leiden University Medical Centre (LUMC), Leiden, the Netherlands; 7 Fondation pour la Recherche Scientifique (FORS), Cotonou, Benin

**Keywords:** malaria, newborn, neonate, treatment, management

## Abstract

**Background:**

Neonatal malaria remains underrecognised despite persistent high neonatal mortality in endemic regions. Evidence to guide diagnosis, classification, and treatment during the first 28 days of life is scarce, and neonatal-specific recommendations are largely absent. We conducted an international survey to describe current practices in neonatal malaria management and identify gaps in care.

**Methods:**

We performed an anonymous online survey of healthcare professionals involved in neonatal malaria care between April 2024 and July 2025. The questionnaire, available in English, French, Portuguese, and Spanish, was distributed through professional networks and targeted invitations, assessing screening practices, diagnostic approaches, disease definitions, treatment strategies, dosing approaches, and perceived challenges. Data were analysed descriptively.

**Results:**

A total of 173 respondents from malaria-endemic and non-endemic settings were included; most (91%) were based in sub-Saharan Africa. Most (61%) reported diagnosing neonatal malaria at least once yearly and 39% at least once a month. Most respondents (72%) did not distinguish between asymptomatic and symptomatic neonatal parasitaemia, and half (49%) did not differentiate between uncomplicated or severe malaria in neonates. The most used drugs were intravenous artesunate and artemether-lumefantrine for oral treatment. Among those using oral therapy, 71% reported using paediatric formulations, while 38% crushed tablets prior to administration. Crushed tablets were mostly mixed with water (90%), followed by expressed breast milk (23%) and infant formula (20%). In a case vignette of neonatal malaria, 87% of respondents recommended inpatient management with initial intravenous artesunate (92%) followed by oral artemisinin-based combination therapy (66%) with treatment duration ranging from 1 to 10 days and follow-up within 28 days (33%).

**Conclusions:**

This survey demonstrates consensus in antimalarial drug selection alongside significant practice variation in disease classification and dosing, highlighting the need for evidence-based guidelines and improved access to age-appropriate formulations for neonatal malaria management.

Despite decades of progress in malaria control, malaria remains an important cause of morbidity and mortality in neonates, particularly in endemic regions [1,2]. Neonatal malaria, defined as malaria occurring within the first 28 days of life, has traditionally been considered rare, largely because of presumed protection from maternal IgG antibodies and foetal haemoglobin [3]. However, emerging evidence suggests that neonatal malaria may be underdiagnosed rather than uncommon [4–6].

A major challenge in improving recognition and management of neonatal malaria is the absence of standardised definitions and guidelines specific to this age group. There is no consensus on whether or not to treat asymptomatic neonatal parasitaemia, nor are there age-specific criteria to differentiate uncomplicated from severe disease in this age group [4,6]. In older patients, the choice between oral and parenteral antimalarial treatment is based on the distinction between uncomplicated and severe malaria; however, in the absence of established severity criteria for neonates, clear guidance for treatment route selection is lacking. Although oral artemisinin-based combinations therapies (ACTs) are also recommended for neonates, newborns have mostly been excluded from clinical trials and the suitability of paediatric drug formulations for neonates has not been assessed [7,8]. As a result, neonates frequently receive ACTs off-label, and the preparation and administration practices of antimalarial treatment for newborns may affect the effectiveness of treatment and be associated with additional risks [9]. Case series have reported a variety of parenteral and oral antimalarial regimens in neonates, including crushed tablets and syrup-based formulations, and in primarily parenterally treated neonates, recommended consecutive oral treatment is not always administered according to guidelines [4].

Because neonatal malaria remains under-recognised, with no dedicated management guidelines and only scant evidence on real-world practice, this survey was designed as an exploratory, descriptive study. Guided by existing malaria recommendations for older infants and children and prior reviews, we focused a priori on three practice domains that are most likely to influence neonatal outcomes:

1. differentiation between asymptomatic and symptomatic parasitaemia,

2. application of severity criteria to guide parenteral *vs.* oral therapy, and

3. dosing strategies and formulations for neonates weighing <5 kg.

The survey aimed to characterise variation in these domains across settings as a basis for identifying priorities for future guideline development and clinical research.

## METHODS

This was an exploratory, cross-sectional survey with descriptive analyses focusing on characterising practice variation within three predefined clinical decision domains (classification, severity-based route selection, and dosing/formulation for neonates <5 kg). The survey targeted healthcare professionals involved in neonatal and malaria care in malaria-endemic and non-endemic settings. A structured, self-administered online questionnaire was developed in English language and subsequently translated into French, Portuguese and Spanish to facilitate participation from malaria-endemic regions. For each language, a native-speaker clinician with experience in paediatrics and/or tropical medicine produced an initial translation. A second bilingual clinician independently reviewed this version, and discrepancies were resolved by consensus. Attention was paid to preserving the meaning of clinical terms (*e.g.* ‘neonate’, ‘uncomplicated malaria’, ‘severe malaria’, ‘rapid diagnostic test’) and to maintaining identical response options across all language versions. A formal forward-back translation procedure was not performed; the structured expert review was used to maximise semantic and conceptual equivalence between translations. The survey was designed to be completed in approximately 10 minutes. In summary, the questionnaire consisted of 21 items organised into four sections: respondent characteristics (profession, years of experience, country of work, type of facility; 5 items), local neonatal care infrastructure and malaria epidemiology (6 items), diagnostic and treatment practices for neonatal malaria (9 items), and an open vignette-based case management question (1 item). Most questions used fixed-choice response formats (single-choice and multiple-choice options), including predefined frequency categories and nominal categories for diagnostic tests and antimalarial drugs. Several questions included an optional free-text field to specify ‘other’ responses, and the final vignette invited a narrative free-text description of management. No Likert-type rating scales were used. The complete questionnaire, including all response options, is provided in Figure S1 in the **Online Supplementary Document**. The clinical vignette (Figure S2 in the **Online Supplementary Document**) described a neonate with uncomplicated *Plasmodium falciparum* malaria and asked respondents to describe management, including level of care, treatment, dosing, duration, and follow-up.

The questionnaire, including the clinical vignette, was reviewed and pilot-tested by members of the target group prior to dissemination to assess clarity, internal consistency, feasibility, and content relevance. The survey was hosted on REDCap and was available from April 2024 to July 2025.

Survey participants were recruited through a mixed strategy: targeted emails using contact details obtained from publications on neonatal malaria, and distribution of the survey link *via* professional networks, including the German Society for Tropical Medicine, Travel Medicine and Global Health (DTG), German Society of Tropical Paediatrics and International Child Health (GTP), International Paediatric Ultrasound Network (IPUN), Centre de Recherches Médicales de Lambaréné (CERMEL), Médecins Sans Frontières (MSF), and additional professional contacts and networks. Because the survey link was openly distributed, the total number of invited clinicians could not be determined.

Data were exported from Redcap and analysed descriptively in Microsoft Excel (Microsoft, Redmond, WA, USA). Categorical variables were presented as frequencies and proportions. For descriptive analyses, respondents were grouped into three malaria-endemic regions (sub-Saharan Africa, South America, and Asia) and one non-endemic category, defined by working in countries without local malaria transmission. We performed a complete-case analysis. We did not impute missing values. Items that were left blank or where respondents selected ‘I don’t know’ were treated as missing for that specific analysis and are reported in the tables as varying denominators (n) where applicable and if not stated differently.

In addition to the prespecified descriptive analysis, an exploratory stratified analysis was performed by facility level (primary, secondary, tertiary) and availability of a formal treatment protocol. Associations between these variables and selected classification and treatment practices were assessed using Pearson χ^2^ test or Fisher exact test where expected cell counts were small. Because questionnaire items were non-mandatory and branching logic led to variable denominators across items, these subgroup analyses were considered exploratory and hypothesis-generating rather than confirmatory. Proportions are reported against item-specific denominator throughout, which are stated alongside each result in the respective tables.

The survey was anonymous, targeted healthcare professionals only, and collected no identifiable information. Ethical approval was therefore not required under local regulations (BO ÄBW §15). Participation was voluntary.

## RESULTS

A total of 178 healthcare professionals completed the survey. Five respondents did not report their country of practice and were therefore excluded, leaving 173 participants included in the analysis. Overall, 143 (83%) respondents worked in malaria-endemic countries. Most respondents were based in sub-Saharan Africa (SSA) (n = 157, 91%), with smaller numbers from South America (SA) (n = 6, 3%), Asia (n = 2, 1%), and non-endemic regions (n = 8, 5%). The geographic distribution of participants is illustrated in **Figure 1**. A list of all countries represented by the survey is provided in Table S1 in the **Online Supplementary Document****.**

**Figure 1:**
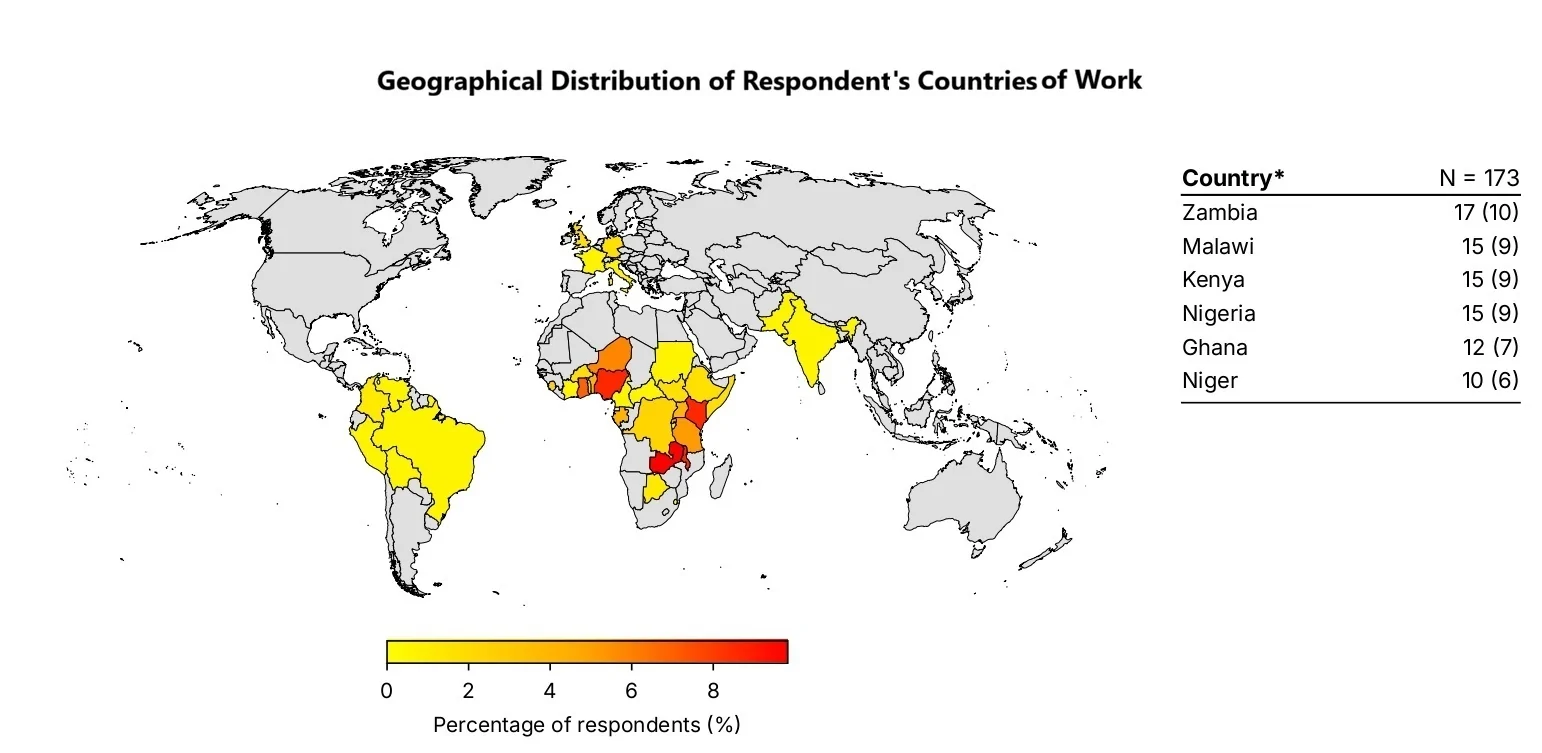
Geographical distribution of respondents’ countries of work with coloured countries being represented by at least one respondent. Colour indicates frequency respondents per country as per colour legend. *Only countries with at least 10 respondents listed in the table.

Most respondents were physicians (n = 113, 65%), the majority of whom had over five years of professional experience (**Table 1**). Among them, medical specialists accounted for 51% (n/N = 58/113), with 45% (n/N = 51/113) being paediatricians or neonatologists (**Table 1**). Nurses (n = 30, 17%) and clinical officers (n = 16, 9%) were also represented.

**Table 1:** Respondent characteristics including medical background and local neonatal care infrastructure

Respondent characteristics, n (%)	Total (n = 173)	SSA (n = 157)	Asia (n = 2)	SA (n = 6)	Non-endemic (n = 8)
Profession of healthcare providers*					
With specialisation	58 (33)	50 (32)	2 (100)	2 (33)	4 (50)
*Paediatrician*	45 (26)	38 (24)	2 (100)	1 (17)	4 (50)
*Neonatologist*	6 (3)	5 (3)	-	1 (17)	-
*ID specialist*	3 (2)	3 (2)	-	-	-
*TM specialist*	2 (1)	2 (1)	-	-	-
*Gynaecologist*	2 (1)	2 (1)	-	-	-
Without specialisation	55 (32)	49 (31)	-	2 (33)	4 (50)
*General physicians*	55 (32)	49 (31)	-	2 (33)	4 (50)
**Non-physician healthcare providers**	60 (35)	58 (37)	-	2 (33)	-
Nurse	30 (17)	30 (19)	-	-	-
Clinical officer	16 (9)	16 (10)	-	-	-
Other†	14 (8)	12 (8)	-	2 (33)	-
**Years of working experience‡**					
<5 years	36 (20)	35 (22)	-	1 (17)	-
5–20	114 (66)	104 (66)	1 (50)	3 (50)	6 (75)
>20	23 (13)	18 (12)	1 (50)	2 (33)	2 (25)
**Local infrastructure**					
Level of care*					
*Primary care*	28 (16)	25 (16)	1 (50)	2 (33)	-
*Secondary*	76 (44)	74 (47)	-	1 (17)	1 (13)
*Tertiary*	60 (35)	51(33)	1 (50)	2 (33)	6 (75)
Newborn care infrastructure*					
*Separate nursery*	34 (20)	32 (20)	1 (50)	-	1 (13)
*Maternity ward*	13 (7)	13 (8)	-	-	-
*Paediatrics without NICU*	28 (16)	26 (17)	-	1 (17)	1 (13)
*Paediatrics with NICU*	90 (52)	81 (52)	1 (50)	2 (33)	6 (75)
Responsible healthcare provider in charge of neonatal malaria care*					
*Physicians*	155 (90)	142 (90)	2 (100)	3 (100)	8 (100)
*Paediatrician*	123 (71)	111 (71)	2 (100)	2 (33)	8 (100)
*General practitioner*	30 (17)	29 (19)	-	1 (17)	-
*Obstetrician*	1 (1)	1 (1)	-	-	-
*Internal medicine*	1 (1)	1 (1)	-	-	-
*Clinical officer*	7 (4)	7 (5)	-	-	-
*Nurse*	4 (2)	4 (3)	-	-	-

ID – infectious disease, TM – tropical medicine, NICU – neonatal intensive care unit

*Profession, level of care, newborn care infrastructure and responsible provider allowed multiple answers; percentages therefore sum to >100%.

†Educator, nutritionist, midwife, public health specialist, medical anthropologist, laboratory-technician, biochemist, general practitioner, and epidemiologist.

‡Years of experience was single choice.

Respondents originated from all levels of healthcare delivery, with a predominance of secondary (n = 76, 44%) and tertiary (n = 60, 35%) care level facilities. Neonatal care was primarily provided within paediatric services with neonatal intensive care units (NICUs) (n = 90, 52%), followed by separate nursery units (n = 34, 20%), paediatric wards without NICU facilities (n = 28, 16%), and lastly maternity wards (n = 13, 7%) (**Table 1**). At respondents’ work settings, neonatal malaria care was overseen predominantly by paediatricians (n = 123, 71%), followed by general practitioners (GP) (n = 30, 17%) (**Table 1**).

Routine malaria screening in asymptomatic neonates was reported by 36 (21%) respondents, whereas 105 (61%) indicated that such screening was not implemented. For diagnosing malaria in neonates, 83 (47%) respondents reported following a protocol; 51 (29%) followed a general diagnostic protocol and 32 (18%) an internal diagnostic protocol. Reported frequencies of testing for and diagnosing malaria in neonates varied geographically (Figures S3–4 in the **Online Supplementary Document**). Overall, 143 (83%) respondents reported regularly testing neonates for suspected malaria, including 39 (23%) testing daily and 96 (56%) testing at least once a month (**Figure 2**). In total, 106 (61%) reported diagnosing neonatal malaria at least once per year, with 15% (n = 26) diagnosing malaria in neonates daily and 39% (n = 67) monthly (**Figure 2**). Of all respondents, 54 (32%) – nearly all from SSA – reported testing for and 65 (38%) reported diagnosing neonatal malaria at least monthly. Rapid diagnostic tests (RDTs) were the most used diagnostic method (n = 125, 72%), followed by microscopy with thick (n = 110, 64%) and thin blood smears (n = 75, 43%).

**Figure 2:**
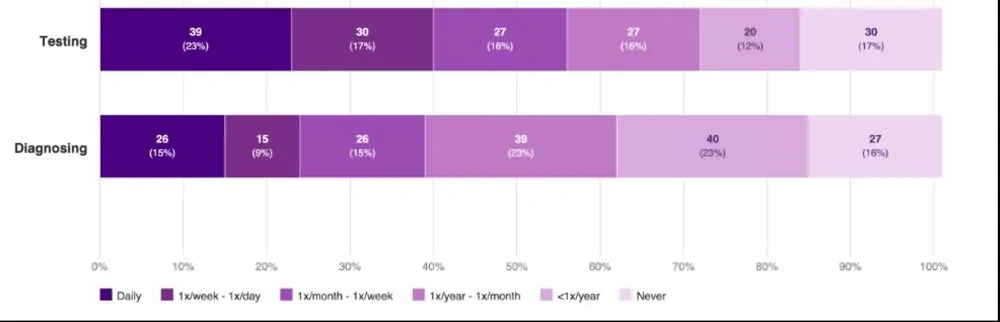
Reported frequency of testing for malaria and diagnosing malaria in neonates including all respondents irrespective of work of practice (n_testing_ = 173 and n_diagnosing_ = 173, respectively), displayed on a share percentage scale.

A total of 130 (75%) respondents reported that mothers of neonates with suspected or confirmed malaria were tested and treated for malaria, while 27 (16%) reported neither screening nor treating mothers for malaria.

Overall, 47 (27%) respondents reported distinguishing between asymptomatic and symptomatic neonatal parasitaemia, whereas the majority (n = 125, 72%) did not make this distinction. Among respondents who provided a definition for asymptomatic parasitaemia (n = 60, 35%), the most common definition (n/N = 32/60, 53%) was laboratory-confirmed parasitaemia in the absence of clinical signs (n/N = 21/32, 66%) but some definitions allowed for mild symptoms (n/N = 5/32, 16%), while others were based on clinical or presumptive diagnosis without laboratory confirmation (n/N = 6/32, 19%). Definitions of symptomatic neonatal malaria were provided by 34 (20%) respondents: most respondents required the presence of both clinical manifestations and laboratory confirmation (n/N = 26/34, 76%); a minority relied on clinical criteria alone or provided non-specific definitions (n/N = 8/34, 24%).

Overall, 87 (50%) respondents reported differentiating between uncomplicated and severe malaria in neonates, whereas 85 (49%) did not make this distinction; one participant did not provide a response. Among those differentiating between uncomplicated and severe malaria in neonates, 52 (30%) respondents reported applying criteria used for older children, whereas 35 (20%) used different criteria. Among those specifying the criteria (n = 48, 86%), WHO or national guidelines were most cited (n/N = 19/48, 40%), followed by clinical criteria (n/N = 18/48, 38%) and laboratory-based classifications (n/N = 11/48, 23%). Definitions of severe neonatal malaria (n = 27, 16%) were primarily based on clinical features (n/N = 15/27, 56%), including convulsions, altered consciousness, lethargy, respiratory distress, poor feeding, and high fever. Combined clinical and laboratory definitions (n/N = 7/27, 26%) included parameters such as hypoglycaemia, severe anaemia, jaundice, metabolic acidosis, or hyperparasitaemia; 3/27 (11%) respondents defined severe neonatal malaria solely based on laboratory thresholds.

Eighteen percent (n/N = 31/173, 18%) of respondents reported known neonatal malaria deaths at their facility, with a median of four deaths (interquartile range (IQR) = 2–10) among those providing estimates.

Treatment protocols for neonatal malaria were available in n/N = 100/173 (58%) of respondents’ facilities; among these, 66/100 (66%) followed general protocols and 34/100 (34%) used internal guidelines. Most (n/N = 138/173, 80%) respondents reported use of parenteral antimalarials with intravenous artesunate being the most frequently used drug (n/N = 128/138, 93%), followed by intravenous quinine (n/N = 25/138, 18%), intramuscular artesunate (n/N = 19/138, 14%), and intramuscular artemether (n/N = 9/138, 7%) (**Table 2**). Rectal antimalarial therapy was also reported, primarily consisting of rectal artesunate (**Table 2**).

**Table 2:** Antimalarial drugs used in neonates

Route of administration	Antimalarial	n (%)
Parenteral (n = 138)*		
	IV artesunate	128 (93)
	IV quinine	25 (18)
	IM artesunate	19 (14)
	IM artemether	9 (6)
Rectal (n = 26)*		
	Rectal artesunate	25 (96)
Oral (n = 80)*		
	Artemether-lumefantrine	69 (85)
	Artesunate-amodiaquine	13 (16)
	Primaquine	5 (6)
	Dihydroartemisinin-piperaquine	2 (3)
	Artesunate-Mefloquine	2 (3)
	Artesunate-Pyronaridine	1 (1)
	Other†	6 (8)

IV – intravenous, IM – intramuscular

*Multiple responses allowed.

†Free text mentions: Amodiaquine & Quinine, Quinine (2x), Amodiaquine (2x), Chloroquine.

Use of oral antimalarial therapy in neonates was reported by n/N = 80/173 (46%) respondents. Artemether-lumefantrine was the most used oral drug (n/N = 69/80, 86%), followed by artesunate-amodiaquine (n/N = 13/80, 16%), and primaquine (n/N = 5/80, 6%). Other ACTs were rarely used (**Table 2**). Among those using oral therapy, 57/80 (71%) reported using paediatric formulations, while 30/80 (38%) crushed tablets prior to administration and 6/80 (8%) administered whole tablets. Crushed tablets were mostly mixed with water (n/N = 27/30, 90%), followed by expressed breast milk (n/N = 7/30, 23%) and infant formula (n/N = 6/30, 20%).

For dosing of oral antimalarials in neonates weighing less than 5 kg, 51/80 (64%) respondents reported calculating doses based on exact body weight, whereas 24/80 (30%) used the same dosing as for children weighing ≥5 kg. A small proportion (5/80, 6%) reported alternative approaches, such as administering half-tablet doses.

A total of 145 respondents addressed the clinical vignette of a 20-day-old neonate with *P. falciparum* parasitaemia. Most (n/N = 126/145, 87%) recommended inpatient management, while outpatient management was rarely (n/N = 3/145, 2%) suggested. The predominant treatment approach involved initiation of intravenous artesunate (n/N = 133/145, 92%), supportive care with intravenous fluids (n/N = 58/145, 40%), and transition to oral ACTs once clinically stable (n/N = 96/145, 66%). Proposed overall treatment duration ranged from 1 to 10 days, clustering most frequently around 7 days, with follow-up visits within 28 days after completion of therapy reported by 48/145 (33%) respondents. 

In exploratory stratified analyses, the proportion of respondents distinguishing asymptomatic from symptomatic neonatal parasitaemia was similar across facility levels, reported by 25% (n/N = 7/28) in primary facilities, 25% (19/75) in secondary facilities, and 28% (n/N = 17/60) in tertiary facilities (*P* = 0.91). Likewise, there was no statistically significant association between facility level and reported use of parenteral antimalarials (n/N = 23/27, 85%, n/N = 60/76, 79%, and n/N = 46/59, 78%, respectively; *P* = 0.728) or intravenous artesunate specifically (n/N = 19/28, 68%, n/N = 55/76, 72%, and n/N = 44/60, 73%, respectively; *P* = 0.862). However, tablet crushing was reported more frequently in tertiary facilities than in primary or secondary facilities (n/N = 15/60, 25% *vs.* n/N = 1/28, 4% and n/N = 12/76, 16%, *P* = 0.042), and the use of a ≥5 kg dosing calculation in neonates weighing <5 kg also differed by facility level (n/N = 14/26, 54% *vs.* n/N = 4/13, 31% and n/N = 6/32, 19%, *P* = 0.019) (Table S2 in the **Online Supplementary Document**). A formal treatment protocol was associated with more structured classification practices. Respondents in facilities with a protocol more often distinguished asymptomatic from symptomatic parasitaemia than those without a protocol (34% (n/N = 34/99) *vs.* 19% (n/N = 12/64), *P* = 0.047) and more often differentiated uncomplicated from severe disease (61% (n/N = 61/100) *vs.* 38% (n/N = 24/64), *P* = 0.005). By contrast, protocol availability was not significantly associated with use of parenteral antimalarials (n/N = 85/100, 85% *vs.* n/N = 47/64, 73%, *P* = 0.105), intravenous artesunate (n/N = 77/100, 77% *vs.* n/N = 41/64, 64%, *P* = 0.105), oral antimalarials (n/N = 49/100, 49% *vs.* n/N = 29/64, 45%, *P* = 0.763), tablet crushing (n/N = 19/100, 19% *vs.* n/N = 11/64, 17%, *P* = 0.932), or use of a ≥5 kg dosing calculation in neonates weighing <5 kg (n/N = 14/46, 30% *vs.* n/N = 9/27, 33%, *P* = 1.000) (Table S2 in the **Online Supplementary Document**).

Respondents highlighted a range of challenges in neonatal malaria management (**Table 3****)**.

**Table 3:** Reported challenges in neonatal malaria care

Management
Lack of neonatal-specific formulation
No syrups, dispersible, or rectal formulations
Limited drug availability
Stock-outs of Artesunate, ACTs, Quinine
Need to crush adult tablets leading to dosing errors
IV Artesunate dosing in newborns leads to wastage
**Diagnostics**
Delayed laboratory results and lack of reliable diagnostic tools
No clear diagnostic algorithms
Often misdiagnosed as neonatal sepsis due to overlapping symptoms
Low index of suspicion because neonatal malaria is rare or “never seen” in many facilities
**Expertise**
Insufficient trained personnel in neonatal malaria management
No neonatal-specific protocols or unclear guidelines
**Resource limitations**
Drug stock-outs
Lack of laboratory equipment/test kits
Financial barriers

## DISCUSSION

This international survey of 173 healthcare professionals, demonstrates substantial variation in neonatal malaria diagnosis, classification, and treatment despite broad agreement on antimalarial drug selection, highlighting the need for neonatal-specific evidence and clinical guidance.

Reported practice was largely concordant with WHO recommendations for antimalarial drug selection in older infants and children, with intravenous artesunate being the predominant parenteral treatment and artemether-lumefantrine the most reported oral therapy. In contrast, substantial heterogeneity was observed in neonatal-specific domains for which no evidence-based guidance exists, including differentiation of asymptomatic from symptomatic parasitaemia, severity classification, and dosing in infants weighing <5 kg. Rather than indicating poor guideline adherence, this variation reflects the absence of neonatal-specific evidence and highlights priorities for future research and guideline development.

The high frequency of neonatal malaria testing and diagnosis reported by respondents challenges the traditional assumption that neonatal malaria is rare. These findings align with emerging evidence suggesting that neonatal malaria is underrecognised rather than uncommon [10,11]. However, reporting bias may have influenced this survey’s outcome, with health professionals regularly caring for neonates with malaria being more inclined to respond to the survey and potentially overrepresenting settings with a high malaria burden. In addition, although 18% of respondents reported known neonatal malaria deaths, a further 12% suspected but could not confirm such deaths, likely reflecting infrequent testing, clinical overlap with neonatal sepsis, and limited routine mortality audit; improved neonatal point-of-care diagnostics and strengthened neonatal death-audit systems could help close this ascertainment gap.

The low proportion of routine screening in asymptomatic neonates (21%), together with the high proportion of respondents who did not distinguish between asymptomatic and symptomatic neonatal malaria (72%), likely reflects the absence of clear guidance on screening indications and the management of asymptomatic parasitaemia. The lack of consensus on whether, or under which circumstances, asymptomatic neonatal parasitaemia should be treated represents a critical gap in clinical guidance, as illustrated by the highly heterogeneous definitions and practices reported by respondents [11]. In addition, persistent uncertainty regarding the balance of potential benefits and harms of treating asymptomatic infections further complicates clinical decision-making. Whereas early treatment may remove a potential contributor to anaemia and impaired growth, withholding treatment might favour the development of partial immunity in high-transmission settings, underscoring the need for context-specific, evidence-based recommendations [12–14]. Notably, nearly half of respondents (49%) reported that they did not distinguish between uncomplicated and severe disease in neonates. This practice pattern likely reflects the absence of validated, age-specific severity criteria for this age group. In older children, the classification of disease severity informs the choice between oral and parenteral antimalarial therapy; however, in the neonatal period, the lack of established severity criteria means that clear, evidence-based guidance on the optimal route of severity-dependent treatment remains unavailable [15].

Exploratory stratified analyses suggest that most reported classification and treatment practices did not differ substantially by facility level, but that the presence of a formal neonatal malaria management protocol may support more structured decision-making. Facilities with a protocol more frequently distinguished asymptomatic from symptomatic parasitaemia and differentiated uncomplicated from severe disease, whereas associations with route of treatment and dosing approaches were less consistent. These subgroup findings, which are hypothesis-generating and constrained by limited sample size and variable denominators, nonetheless point to protocol development and implementation as a potentially important lever to improve standardisation of neonatal malaria care across diverse settings.

Compared with published case series, respondents more frequently reported ACT-based treatment consistent with current recommendations [4], although the high use of intravenous therapy probably reflects uncertainty regarding neonatal severity assessment and limited availability of suitable oral formulation [16,17].

Oral treatment remains challenging because neonatal formulations are largely unavailable. Although most respondents reported using paediatric formulations, tablet crushing remained common and may compromise dosing accuracy and bioavailability. Almost one-third of respondents applied dosing regimens intended for infants weighing 5 kg to neonates (who likely all weighed <5 kg). This pragmatic approach aligns with WHO recommendations for only select oral ACTs, pharmacokinetic data are urgently needed for neonates <5 kg, as distinct neonatal metabolic pathways may require age-specific dosing strategies that cannot be reliably extrapolated from older children [18].

Variation in treatment duration and follow-up within the clinical vignette further illustrates the absence of standardised neonatal management recommendations. Although most respondents recommended intravenous artesunate follow by oral ACTs, optimal treatment duration and follow-up remain uncertain.

Furthermore, the development of international consensus guidelines specifically addressing neonatal malaria management would provide much-needed standardisation of care. Based on our findings, such guideline development should prioritise three areas. First, standardised, neonate-specific case definitions distinguishing asymptomatic from symptomatic parasitaemia are needed. Second, validated severity criteria tailored to neonates are required to inform the choice between oral and parenteral antimalarial therapy. Third, guidelines should be coupled with accelerated regulatory and implementation pathways for paediatric antimalarial formulations suitable for infants under 5 kg, building on emerging pharmacokinetic data for artemether–lumefantrine in small infants [18]. Without age-appropriate formulations, clinicians will continue to depend on tablet crushing and improvised administration methods, which our survey shows remain common practice.

Several limitations merit consideration. First, selection bias may have occurred through targeted recruitment, potentially overrepresenting healthcare professionals with specific interest or expertise in neonatal malaria, while under-representing providers in primary and secondary facilities without specialised neonatal services; as a result, the findings reflect practice among an engaged, better-resourced subgroup and may not be generalisable to all settings where neonatal malaria occurs. Non-response bias cannot be quantified, as the denominator of invited clinicians is unknown and only fully completed questionnaires were retained. The sample is therefore likely skewed towards providers with particular interest in neonatal malaria and/or higher diagnostic activity, which may limit the generalisability of the findings to all healthcare professionals in endemic settings. Second, the survey relied on self-reported practices rather than direct observation, which may be subject to recall and social desirability bias. Third, the predominance of respondents from SSA, though reflecting the geographic distribution of malaria burden, limits generalisability to other endemic regions. Fourth, although the questionnaire was translated into French, Portuguese and Spanish using a structured process involving native-speaker clinicians and independent review, we did not conduct formal back-translation; therefore, some residual differences in wording across language versions and the resulting measurement error cannot be fully excluded. Fifth, we could not determine the total number of clinicians who received the survey invitation, particularly for the invitations distributed *via* professional networks and mailing lists. This precluded calculation of a response rate and a quantitative assessment of non-response bias. Furthermore, the survey’s dosing questions were restricted to broad strategies (use of exact weight-based dosing *vs. *≥ 5 kg thresholds) and did not capture the specific numerical doses, dosing tables or pharmacokinetic considerations underlying individual prescriptions; the results therefore indicate substantial heterogeneity and potential dosing risk but cannot quantify the magnitude of over- or underdosing relative to model-based or trial-derived recommendations in neonates <5 kg. Lastly, the anonymous nature of the survey precluded verification of responses or follow-up for clarification. Notably, the inclusion of a case vignette represents a particular strength of this study, offering a concrete and realistic scenario to explore clinical decision-making; future studies may build on this by incorporating a broader range of presentations to capture the full spectrum of neonatal malaria management challenges.

## CONCLUSIONS

In conclusion, the survey suggests that unstructured classification, inconsistent severity-based route selection, and highly variable dosing/formulation practices in neonates <5 kg together translate into risks of both overtreatment and undertreatment, misaligned oral *vs.* parenteral therapy, and clinically important under- or overdosing, highlighting classification of parasitaemia, severity-based treatment route selection, and dosing/formulation for neonates <5 kg as priority targets for neonatal-specific guidelines, improved formulations, and implementation support.

## Additional material


Online Supplementary Document

## Data Availability

**Data availability:** Data will be provided by the authors upon request.
