# Supplementary material for: Neonatal malaria management: a global survey of current practice
Source: J Glob Health. 2026 Nov 6;16:04367. doi: 10.7189/jogh.16.04367 (PMC13741790; doi:10.7189/jogh.16.04367)
Supplement: Supplementary file 1 [file jogh-16-04367-s001.pdf]

Supplement to: Sultanli A, Remppis J, Honkpehedji YJ, Reif C, Esen M, Akim Adegnika A, Bélard S. Neonatal malaria management: a global survey of current practice. J Glob Health. 2026;16:04367.

Figure S1 – Survey

International Anonymous Survey on Malaria Management in Newborns: Insights from Healthcare Professionals

Thank you for agreeing to take part in this survey.

This survey seeks to compile diagnostic methodologies and treatment strategies used by healthcare professionals in addressing malaria in newborns within their first month of life.

Below, you will find a maximum of 21 questions. The expected time to complete the survey is approximately 10 minutes.

Your answers will remain completely anonymous throughout the survey.

Note: neonates = newborns in their first month of life.

Preferred language

◯ English
◯ French
◯ Portuguese
◯ Spanish

---

1. What is your profession?

Multiple answers possible.

☐ Nurse
☐ Clinical Officer
☐ Medical Doctor
☐ Paediatrician
☐ Neonatologist
☐ Gynecologist
☐ Infectious Disease Specialist
☐ Tropical Medicine Specialist
☐ Other

Please specify "other": ______________________________

---

2. How many years of working experience do you have?

◯ < 5 years
◯ 5-20 years
◯ >20 years

3. In which country do you work?

◯ Afghanistan
◯ Albania
◯ Algeria
◯ Andorra
◯ Angola
◯ Antigua & Deps
◯ Argentina
◯ Armenia
◯ Australia
◯ Austria
◯ Azerbaijan
◯ Bahamas
◯ Bahrain
◯ Bangladesh
◯ Barbados
◯ Belarus
◯ Belgium
◯ Belize
◯ Benin
◯ Bhutan
◯ Bolivia
◯ Bosnia Herzegovina
◯ Botswana
◯ Brazil
◯ Brunei
◯ Bulgaria
◯ Burkina
◯ Burundi
◯ Cambodia
◯ Cameroon
◯ Canada
◯ Cape Verde
◯ Central African Rep
◯ Chad
◯ Chile
◯ China
◯ Colombia
◯ Comoros
◯ Congo
◯ Congo {Democratic Rep}
◯ Costa Rica
◯ Croatia
◯ Cuba
◯ Cyprus
◯ Czech Republic
◯ Denmark
◯ Djibouti
◯ Dominica
◯ Dominican Republic
◯ East Timor
◯ Ecuador
◯ Egypt
◯ El Salvador
◯ Equatorial Guinea
◯ Eritrea
◯ Estonia
◯ Ethiopia
◯ Fiji
◯ Finland
◯ France
◯ Gabon
◯ Gambia
◯ Georgia
◯ Germany
◯ Ghana
◯ Greece
◯ Grenada
◯ Guatemala

◯ Guinea
◯ Guinea-Bissau
◯ Guyana
◯ Haiti
◯ Honduras
◯ Hungary
◯ Iceland
◯ India
◯ Indonesia
◯ Iran
◯ Iraq
◯ Ireland {Republic}
◯ Israel
◯ Italy
◯ Ivory Coast
◯ Jamaica
◯ Japan
◯ Jordan
◯ Kazakhstan
◯ Kenya
◯ Kiribati
◯ Korea North
◯ Korea South
◯ Kosovo
◯ Kuwait
◯ Kyrgyzstan
◯ Laos
◯ Latvia
◯ Lebanon
◯ Lesotho
◯ Liberia
◯ Libya
◯ Liechtenstein
◯ Lithuania
◯ Luxembourg
◯ Macedonia
◯ Madagascar
◯ Malawi
◯ Malaysia
◯ Maldives
◯ Mali
◯ Malta
◯ Marshall Islands
◯ Mauritania
◯ Mauritius
◯ Mexico
◯ Micronesia
◯ Moldova
◯ Monaco
◯ Mongolia
◯ Montenegro
◯ Morocco
◯ Mozambique
◯ Myanmar, {Burma}
◯ Namibia
◯ Nauru
◯ Nepal
◯ Netherlands
◯ New Zealand
◯ Nicaragua
◯ Niger
◯ Nigeria
◯ Norway
◯ Oman
◯ Pakistan
◯ Palau
◯ Panama
◯ Papua New Guinea
◯ Paraguay
◯ Peru
◯ Philippines

- ◯ Poland
- ◯ Portugal
- ◯ Qatar
- ◯ Romania
- ◯ Russian Federation
- ◯ Rwanda
- ◯ St Kitts & Nevis
- ◯ St Lucia
- ◯ Saint Vincent & the Grenadines
- ◯ Samoa
- ◯ San Marino
- ◯ Sao Tome & Principe
- ◯ Saudi Arabia
- ◯ Senegal
- ◯ Serbia
- ◯ Seychelles
- ◯ Sierra Leone
- ◯ Singapore
- ◯ Slovakia
- ◯ Slovenia
- ◯ Solomon Islands
- ◯ Somalia
- ◯ South Africa
- ◯ South Sudan
- ◯ Spain
- ◯ Sri Lanka
- ◯ Sudan
- ◯ Suriname
- ◯ Swaziland
- ◯ Sweden
- ◯ Switzerland
- ◯ Syria
- ◯ Taiwan
- ◯ Tajikistan
- ◯ Tanzania
- ◯ Thailand
- ◯ Togo
- ◯ Tonga
- ◯ Trinidad & Tobago
- ◯ Tunisia
- ◯ Turkey
- ◯ Turkmenistan
- ◯ Tuvalu
- ◯ Uganda
- ◯ Ukraine
- ◯ United Arab Emirates
- ◯ United Kingdom
- ◯ United States
- ◯ Uruguay
- ◯ Uzbekistan
- ◯ Vanuatu
- ◯ Vatican City
- ◯ Venezuela
- ◯ Vietnam
- ◯ Yemen
- ◯ Zambia
- ◯ Zimbabwe

---

4. Are you working in a malaria endemic area?

- ◯ No
- ◯ Yes

5. What level of healthcare facility are you working at?

Multiple answers possible.

☐ Primary care (community health centres, general practitioners)
☐ Secondary care (clinics and hospitals)
☐ Tertiary care (specialised medical centers)
☐ Other

Please specify "other":

____

6. At your facility, where are neonates cared for?

Multiple answers possible.

☐ In the paediatric ward WITH a dedicated neonatology/neonatal intensive care unit (NICU)
☐ In the paediatric ward WITHOUT a dedicated neonatology/NICU
☐ In the maternity ward
☐ In a separate nursery unit
☐ Other

Please specify "other":

____

7. Who oversees the care of neonatal malaria at your facility?

Multiple answers possible.

☐ Paediatricians
☐ General Practitioner
☐ Internal Medicine Specialist
☐ Obstetrician/Gynecologist
☐ Clinical Officer
☐ Nurse
☐ Other

Please specify "other":

____

8. How often are blood tests for malaria performed in neonates at your facility?

◯ Never
◯ < 1x/year
◯ 1x/year to 1x/month
◯ 1x/month to 1x/week
◯ 1x/week to 1x/day
◯ Daily

9. How often is malaria diagnosed in neonates at your facility?

◯ Never
◯ < 1x/year
◯ 1x/year to 1x/month
◯ 1x/month to 1x/week
◯ 1x/week to 1x/day
◯ Daily

10. In your institution, what is the ratio of neonates diagnosed with neonatal malaria to the total number of births?

____

11. Which of the following tests are performed in neonates suspected of malaria at your facility?

Multiple answers possible.

☐ Clinical Assessment
☐ Rapid Diagnostic Test (RDT)
☐ Microscopy - thick blood smear
☐ Microscopy - thin blood smear
☐ Other

Please specify "other":

____

12. Are mothers of neonates suspected and diagnosed with malaria also addressed?

◯ No, neither screening for malaria nor treatment for malaria is done in the mother
◯ Yes, the mother is screened for malaria using laboratory tests and treated if the tests return positive for malaria
◯ Yes, the mother is always treated for malaria regardless of tests and tests results
◯ In a neonate suspected or diagnosed with malaria, only the mother is treated with antimalarials
◯ Other

Please specify "other":

____

13. Do you differentiate between asymptomatic and symptomatic parasitaemia in neonatal malaria cases in your facility?

◯ No
◯ Yes

Please provide the case definition you use for asymptomatic parasitaemia in neonates:

Please provide the case definition you use for symptomatic parasitaemia in neonates:

14. How do you determine the severity of neonatal malaria?

◯ We do not distinguish between uncomplicated and severe malaria in neonates
◯ We use the same criteria as for older patients to distinguish between uncomplicated and severe malaria in neonates
◯ We distinguish between uncomplicated and severe malaria in neonates using different criteria than those for older patients

---

Specify your reference criteria for older patients:

Specify your criteria for severe malaria in neonates:

---

15. At your facility, has neonatal malaria caused deaths in neonates?

◯ Yes, I am aware of neonatal deaths caused by malaria
◯ Yes, I think that neonates have died of malaria
◯ No, I am unaware of any neonatal deaths caused by malaria
◯ I don't know

How many deaths related to neonatal malaria do you recall during the past year?

____

Are neonates presenting at your facility routinely screened for malaria independent of their clinical presentation?

◯ Yes, neonates are routinely screened for malaria independent of their clinical presentation
◯ No, neonates are not routinely screened for malaria

What diagnostic protocols are followed at your facility for suspected neonatal infection in sick newborns ?

◯ There is no standard protocol that is followed at my facility
◯ Internal protocol/SOP, specify
◯ General protocol/SOP, specify
◯ Other

Please specify 'internal protocol/SOP':

____

Please specify "general protocol/SOP":

____

Please specify "other":

____

16. What treatment protocols are followed at your facility for the management of malaria in neonates?

◯ There is no standard protocol that is followed at my facility.
◯ Internal protocol/SOP, specify
◯ General protocol/SOP, specify
◯ Other

Please specify "internal protocol/SOP":

Please specify "general protocol/SOP":

Please specify "other":

17. Do you use parenteral antimalarial drugs to treat malaria in neonates?

◯ No
◯ Yes

Which drugs do you use?

Multiple answers possible.

☐ Intravenous artesunate
☐ Intramuscular artesunate
☐ Intramuscular artemether
☐ Intravenous quinine
☐ Other

Please specify "other":

18. Do you use rectal antimalarial drugs to treat malaria in neonates?

◯ No
◯ Yes

Which rectal antimalarials do you use?

Multiple answers possible.

☐ Rectal artesunate
☐ Other

Please specify "other":

____

19. Do you use oral antimalarial drugs to treat malaria in neonates?

◯ No
◯ Yes

Which oral drugs do you use?

Multiple answers possible.

☐ Artemether-Lumefantrine
☐ Dihydroartemisinin-Piperaquine
☐ Artesunate-Mefloquin
☐ Artesunate-Amodiaquin
☐ Artesunate-Pyronaridine
☐ Primaquine
☐ Other

Please specify "other":

How do you administer oral antimalarials to the neonates ?

Multiple answers possible.

☐ As a whole tablet
☐ We use paediatric drug formulations (e.g., syrup, dispersible tablet, granule), specify
☐ We crush tablets

Please specify the paediatric formulation, and if reconstitution is needed, with what you reconstitute:

What target dose of the oral antimalarial do you administer to neonates weighing less than 5 kg body weight?

◯ We administer an antimalarial dose calculated to the precise body weight of the neonate
◯ We administer the same antimalarial dose as for children weighing 5 kg body weight
◯ Other

Please specify "other":

____

How do you administer crushed tablets to neonates?

Multiple answers possible.

☐ Water
☐ Pumped breast milk
☐ Baby milk
☐ Other

Please specify "other":

20. Which challenges in managing neonatal malaria do you face in your facility?

Figure S2 - Case Vignette

21. This is a case from a malaria-endemic area. Please answer the following questions on how you would manage this case at your facility.

Name: Baby Liam
Age: 20 days old
Gender: Male
Weight: 3.100 kg
Haemoglobin: 13.5 g/dL

Baby Liam is brought to your care facility presenting with refusal to feed, excessive crying, irritability, and a rectal temperature of 38° Celcius. Microscopy is positive for P. falciparum malaria with a parasitaemia of 3000 µ/L.

How would you manage this neonate?

Please describe, as precisely as possible, management including:

- in/outpatient care
- choice of drugs including
- dosage
- route
- regime
- duration of treatment
- follow-up

Table S1 – List of Countries Answering the Survey

Working Country
Benin
Bolivia
Botswana
Brazil
Burkina
Burundi
Cameroon
Central African Rep
Colombia
Congo {Democratic Rep}
Ethiopia
France
Gabon
Gambia
Germany
Ghana
India
Italy
Ivory Coast
Kenya
Malawi
Niger
Nigeria
Pakistan
Peru
Rwanda
Sierra Leone
Somalia
South Sudan
Sudan
Swaziland
Tanzania
Togo
Uganda
United Kingdom
Venezuela
Zambia

Table S2. Classification and treatment practices stratified by facility level and treatment-protocol availability

By facility level

Practice	Primary (n=28)	Secondary (n=76)	Tertiary (n=60)	p-value
Distinguishes asymptomatic from symptomatic parasitaemia	7/28 (25%)	19/75 (25%)	17/60 (28%)	0.910
Differentiates uncomplicated from severe disease	17/27 (63%)	42/76 (55%)	24/60 (40%)	0.082
Uses parenteral antimalarials	23/27 (85%)	60/76 (79%)	46/59 (78%)	0.728
Uses intravenous artesunate	19/28 (68%)	55/76 (72%)	44/60 (73%)	0.862
Uses oral antimalarials	13/27 (48%)	33/76 (43%)	30/59 (51%)	0.685
Crushes tablets for administration	1/28 (4%)	12/76 (16%)	15/60 (25%)	0.042 (Fisher)
Applies ≥5 kg dosing threshold for neonates <5 kg	4/13 (31%)	6/32 (19%)	14/26 (54%)	0.019 (Fisher)

By treatment-protocol availability

Practice	Protocol (n=100)	No protocol (n=64)	p-value
Distinguishes asymptomatic from symptomatic parasitaemia	34/99 (34%)	12/64 (19%)	0.047
Differentiates uncomplicated from severe disease	61/100 (61%)	24/64 (38%)	0.005
Uses parenteral antimalarials	85/100 (85%)	47/64 (73%)	0.105
Uses intravenous artesunate	77/100 (77%)	41/64 (64%)	0.105
Uses oral antimalarials	49/100 (49%)	29/64 (45%)	0.763
Crushes tablets for administration	19/100 (19%)	11/64 (17%)	0.932
Applies ≥5 kg dosing threshold for neonates <5 kg	14/46 (30%)	9/27 (33%)	1.000 (Fisher)

Tests of association are χ^2 unless marked: (Fisher) = Fisher exact test, applied where any expected cell count was <5. Denominators vary because individual items were not mandatory and the questionnaire used branching logic; the dosing-threshold item was answered only by respondents using oral antimalarials in neonates <5 kg. The study was not powered for these subgroup comparisons, and results are hypothesis-generating.

Figure S3 - Reported frequencies of testing for malaria in neonates per country

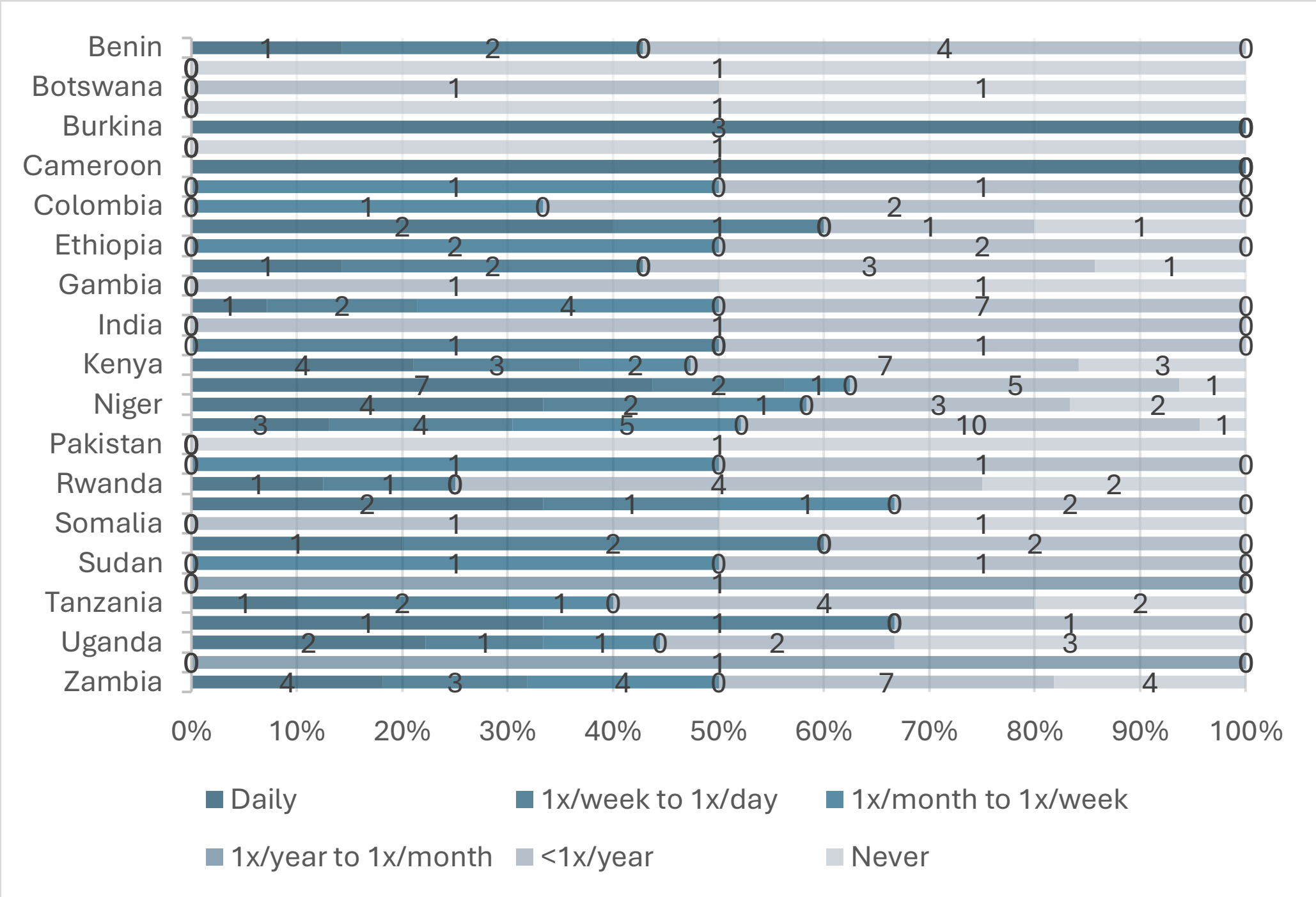

Figure S3. Reported frequencies of testing for malaria in neonates per country (malaria-endemic countries only, alphabetical order).

Figure S4 - Reported frequencies of diagnosing malaria in neonates per country.

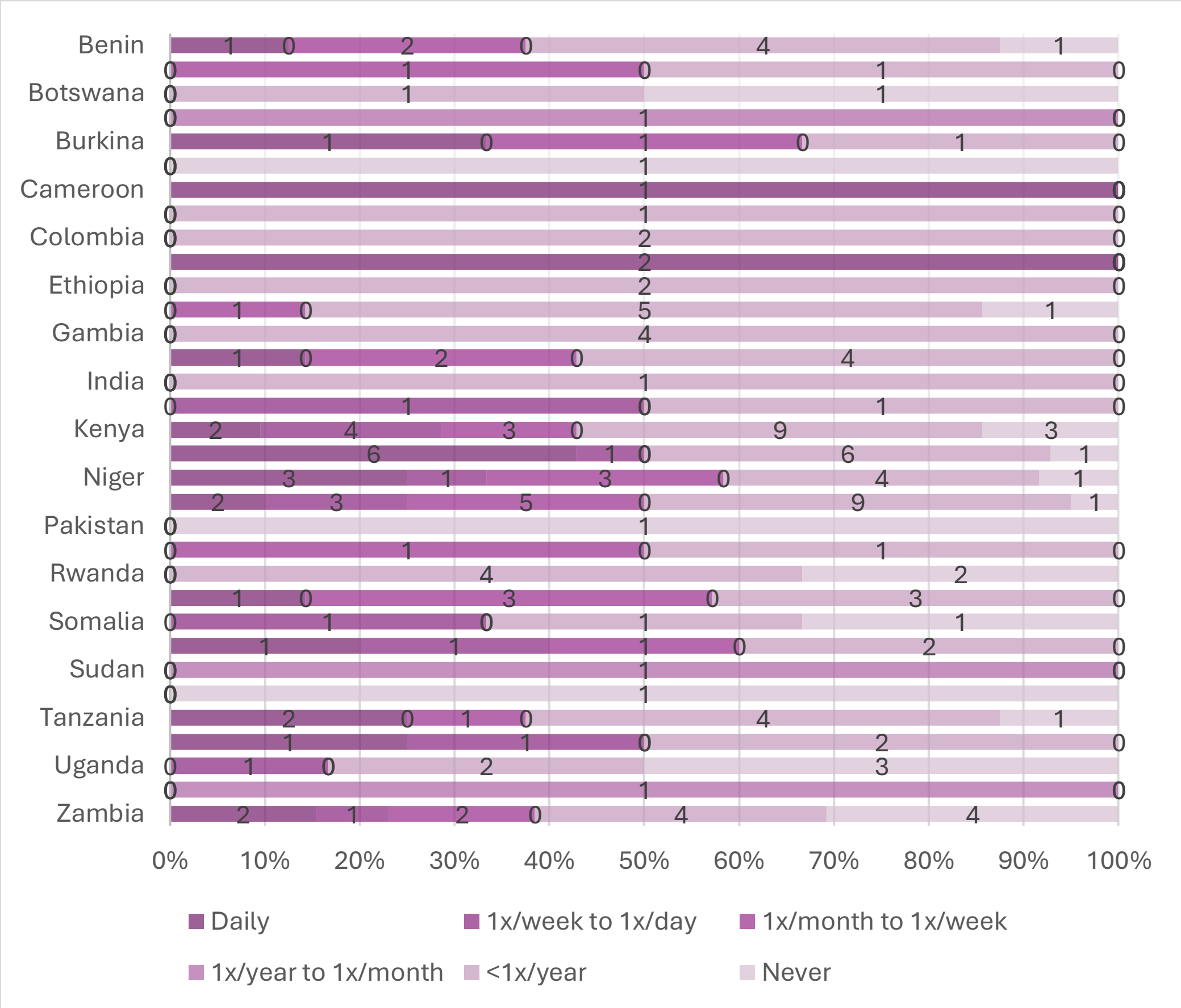

Figure S4. Reported frequencies of diagnosing malaria in neonates per country (malaria-endemic countries only, alphabetical order).
